# Optimal sampling strategies for darunavir and external validation of the underlying population pharmacokinetic model

**DOI:** 10.1007/s00228-020-03036-2

**Published:** 2020-11-11

**Authors:** Gabriel Stillemans, Leila Belkhir, Bernard Vandercam, Anne Vincent, Vincent Haufroid, Laure Elens

**Affiliations:** 1 Integrated PharmacoMetrics, PharmacoGenomics and PharmacoKinetics, Louvain Drug Research Institute, Université catholique de Louvain, Avenue E. Mounier 72, B01.72.0, Brussels, Belgium; 2 Louvain Centre for Toxicology and Applied Pharmacology, Institut de recherche expérimentale et clinique, Université catholique de Louvain, Brussels, Belgium; 3 AIDS Reference Center, Department of Internal Medicine, Cliniques universitaires Saint-Luc, Université catholique de Louvain, Brussels, Belgium; 4 Department of Clinical Chemistry, Cliniques universitaires Saint-Luc, Brussels, Belgium

**Keywords:** Population pharmacokinetics, Darunavir, HIV, External validation, Optimal sampling strategy, Limited sampling strategy

## Abstract

**Purpose:**

A variety of diagnostic methods are available to validate the performance of population pharmacokinetic models. Internal validation, which applies these methods to the model building dataset and to additional data generated through Monte Carlo simulations, is often sufficient, but external validation, which requires a new dataset, is considered a more rigorous approach, especially if the model is to be used for predictive purposes. Our first objective was to validate a previously published population pharmacokinetic model of darunavir, an HIV protease inhibitor boosted with ritonavir or cobicistat. Our second objective was to use this model to derive optimal sampling strategies that maximize the amount of information collected with as few pharmacokinetic samples as possible.

**Methods:**

A validation dataset comprising 164 sparsely sampled individuals using ritonavir-boosted darunavir was used for validation. Standard plots of predictions and residuals, NPDE, visual predictive check, and bootstrapping were applied to both the validation set and the combined learning/validation set in NONMEM to assess model performance. D-optimal designs for darunavir were then calculated in PopED and further evaluated in NONMEM through simulations.

**Results:**

External validation confirmed model robustness and accuracy in most scenarios but also highlighted several limitations. The best one-, two-, and three-point sampling strategies were determined to be pre-dose (0 h); 0 and 4 h; and 1, 4, and 19 h, respectively. A combination of samples at 0, 1, and 4 h was comparable to the optimal three-point strategy. These could be used to reliably estimate individual pharmacokinetic parameters, although with fewer samples, precision decreased and the number of outliers increased significantly.

**Conclusions:**

Optimal sampling strategies derived from this model could be used in clinical practice to enhance therapeutic drug monitoring or to conduct additional pharmacokinetic studies.

**Supplementary Information:**

The online version contains supplementary material available at 10.1007/s00228-020-03036-2.

## Introduction

We previously developed a population pharmacokinetic (PK) model for darunavir (DRV), an HIV-1 protease inhibitor used in antiretroviral therapy (ART), based on data collected in a large cohort of ambulatory patients using cobicistat- (COB) or ritonavir- (RTV) boosted DRV [1]. DRV PK are characterized by major inter-individual variability. Among investigated covariates, those that contributed the most to this variability were alpha-1 acid glycoprotein (AAG), sex, CYP3A5*3, and *SLCO3A1* rs8027174 variants. This PK model was used to conduct simulations of alternative dosage regimens, including a reduction of the daily dose from 800 mg q24h to 600 mg q24h, 400 mg q24h, or 800 mg 5 days in a row followed by 2 days of treatment interruption. The model was evaluated and validated using standard goodness of fit metrics (precision, shrinkage) and graphical methods (plots of predictions and residuals), normalized prediction distribution errors (NPDE), and bootstrapping. These techniques are considered sufficient in most cases, but so-called external validation methods, which require a new dataset, remain the superior approach when one wishes to ensure the quality of model predictions, which is paramount when this model is to be used for predictive purposes [2]. To provide additional information regarding the accuracy and robustness of our model, we decided to perform external validation using a new dataset. Based on this model, we will also derive optimal sampling strategies (OSSs) that would allow one to obtain accurate estimates of PK parameters with as few samples as possible. A single concentration, such as the trough concentration (C_0_), may not be sufficient to reliably assess individual exposure, but as the number of collected samples increases, so do laboratory and personnel costs, not to mention the inconvenience it represents for the patient. Therefore, an OSS is necessary to strike a balance between accuracy and convenience/feasibility, both in terms of the optimal number of samples and the optimal timing of sample collection. One method for obtaining OSSs is based on multiple regression models [3]. However, these have several limitations: they are not PK models and usually only predict one variable (e.g., AUC), they do not explicitly take inter- and intra-individual variability into account, they cannot be used to extrapolate to different types of patients, and the OSS can only include timepoints that were included in the original design. Bayesian methods are a more elegant and efficient approach that has been introduced to population PK. Among them, D-optimal design, which is based on optimization of the Fisher information matrix, is the most frequently employed [4, 5]. DRV OSSs will be calculated according to a D-optimal design and then further evaluated through Monte Carlo simulations.

## Materials and methods

### Population PK model

The model used throughout the present paper has been extensively described in a previous publication [1]. Briefly, this model was developed using NONMEM (version 7.4.3) [6] based on a dataset comprising 127 HIV-positive patients (learning set). DRV PK was described by a one-compartment model with first-order absorption and first-order elimination. The final model included the following covariates: AAG, sex, CYP3A5*3, and *SLCO3A1* rs8027174. A prior distribution had been set on all fixed effects and inter-individual random effects, based on a subset of rich pharmacokinetic data.

### Validation dataset

The validation set was composed of data collected between November 2012 and April 2016 at the Cliniques universitaires Saint-Luc (Brussels, Belgium) as part of a study by Belkhir et al. [7]. Original approval for data collection was granted by the local ethics committee (Comité d’Ethique Hospitalo-Facultaire des Cliniques Saint-Luc, approval number B403201214460). One hundred eighty DRV plasma concentrations were measured in 164 individuals (one sample per dose interval) using the same liquid chromatography-diode array detector method that was used for the learning set [8]. One concentration was excluded because the post-intake time was unknown. Genotypes were unavailable in 18 subjects (11.0%), whereas AAG was not available at all. Genetic covariates were assumed to be missing at random for baseline covariate analysis and then substituted by the most frequent genotype for the patient’s race for PK modeling and simulation. Drug-drug interactions that could alter DRV concentrations were summarized for each patient by the presence or absence of at least one drug belonging to each of the following groups: CYP3A inhibitors, CYP3A inducers, and P-glycoprotein inhibitors, using the methodology outlined in our previous publication [1]. There was some patient overlap between the datasets: 51 individuals contributed data to both studies. Baseline covariates were compared using Fisher’s exact test for categorical covariates or Wilcoxon’s rank-sum test for continuous ones. Statistical analyses were conducted in R (version 3.5.2) [9].

### External validation

An external validation procedure similar to the one used in Krekels et al. [10] was applied. First, the model was refitted to the validation data with all parameters fixed to their final estimates, Bayesian estimation was performed, and the goodness of fit was assessed. PsN (version 4.7.0) was used for handling NONMEM runs [11]. NPDE, which are a more appropriate metric for validation compared to standard weighted residuals, were computed for the validation set based on 1000 simulations from the final model, by means of the npde package (version 2.0) [12–14]. Their distribution was compared to the reference distribution using graphical methods and appropriate statistical tests: the Wilcoxon signed-rank test (H_0_: mean = 0), Fisher variance test (H_0_: variance = 1), and Shapiro-Wilk test (H_0_: normal distribution). Additionally, a prediction-corrected visual predictive check (pcVPC) was constructed from these 1000 simulations, and the observed concentrations from the validation set were overlaid on the prediction intervals. Finally, the validation and learning sets were merged, and the model refitted to this combined dataset. For subjects who contributed data to both studies, their data points were considered separate occasions. 95% CIs were computed from 1000 bootstrapped datasets.

### Optimal sampling strategy

Sampling strategies were evaluated according to the D-optimality criterion in PopED for R (version 0.4.0) using a sequential combination of adaptive random search, line search, and BFGS algorithms [15, 16]. Because both the learning and the validation set were primarily composed of PK data collected at random timepoints, there was no initial design in the traditional sense of the word (as in, a specific set of timepoints for observations). Consequently, it was not possible to evaluate and optimize it using D-optimality without making strong assumptions. However, the subset of rich PK data, which was originally used to define a prior distribution on all parameters, could be used as a substitute for the full learning set. Therefore, the initial design in PopED mirrored the rich PK substudy in terms of measured parameters, sample size, sampling scheme, and dose regimens: 12 patients treated with DRV 800 mg q24h, sampled 8 times over 6 h (C_0_, C_0.5_, C_1_, C_2_, C_3_, C_4_, C_5_, C_6_) at PK steady-state. Designs could include up to four points, sampling times were constrained to be identical in all subjects, and other aspects of the design (e.g., sampling density, population size, dose regimens) were kept fixed. Sensitivity analysis was conducted by perturbing each fixed effect by ± 30%, one at a time.

To further evaluate these strategies, the final model in NONMEM was used to perform Monte Carlo simulations in order to generate a new virtual population. Two hundred fifty PK profiles were generated for each subject from the learning set, using their baseline covariate values to derive their PK parameters. The reference area under the curve over 24 h (AUC_ref_) was obtained from each individual’s daily dose and clearance. Individual Bayesian estimates of clearance were then computed using the timepoints selected in PopED (rounded to the nearest discrete value to obtain near-optimal timepoints), and the corresponding AUCs were derived (AUC_OSS_). The performance of each strategy was assessed by calculating Spearman’s ρ between AUC_ref_ and AUC_OSS_ for all *n* subjects, as well as prediction errors (PEs), the mean percentage error (MPE), and the root mean square percentage error (RMSPE) using the following equations:

$$ \mathrm{PE}=\frac{{\mathrm{AUC}}_{\mathrm{ref}}-{\mathrm{AUC}}_{\mathrm{OSS}}}{{\mathrm{AUC}}_{\mathrm{ref}}} $$

$$ \mathrm{MPE}=100\%\times \frac{1}{n}\sum PE $$

$$ \mathrm{RMSPE}=100\%\times \sqrt{\frac{1}{n}\sum {PE}^2} $$

Values of 15% RMSPE were considered acceptable [3, 17]. Additionally, the proportion of AUCs predicted within ± 15% of the reference value was computed.

## Results

### Datasets

**Fig. 1:**
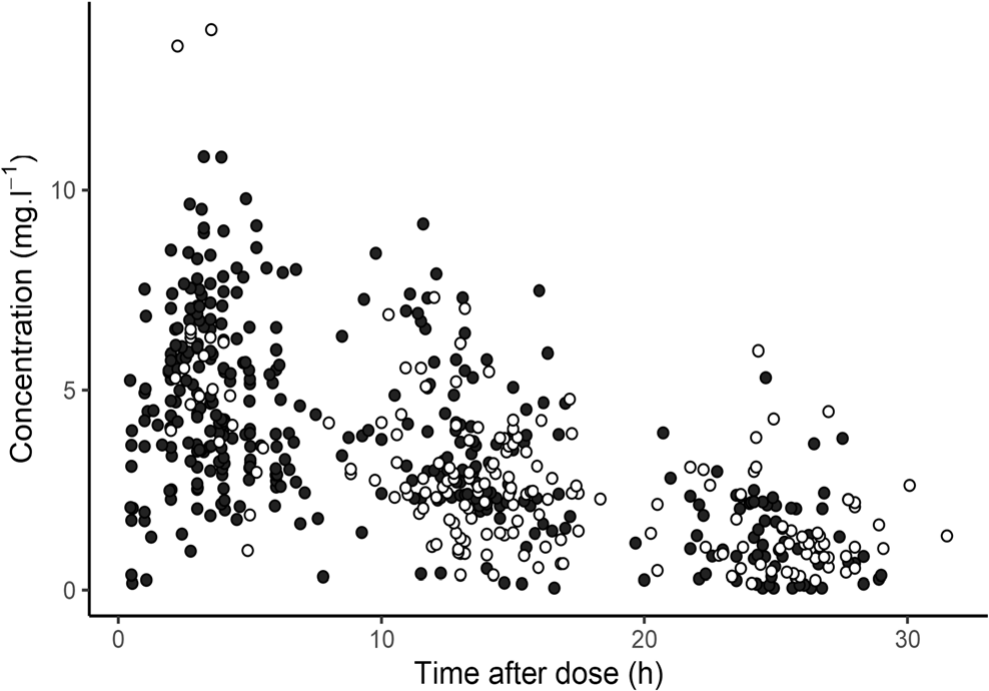
Pharmacokinetic data in the learning and validation sets. Observations from the learning set are represented with black circles and validation set with white circles

Summary statistics of the learning and validation sets are provided in Table 1. All subjects in the validation set were using RTV-boosted DRV since this data was collected prior to the commercialization of COB, while subjects in the learning set used COB-boosted DRV for the most part (85.8% of subjects). In both cases, DRV 800 mg q24h was the most frequent dosage (91.3% and 67.1% of individuals in the learning and validation sets, respectively), followed by 600 mg q12h (7.9% and 29.3%). Concomitant antiretrovirals differed significantly, in part due to the different time frames in which the two studies were conducted: the most frequent combinations in the learning set were lamivudine/dolutegravir/DRV (18.1%), tenofovir/emtricitabine/DRV (18.1%), and dolutegravir/DRV (17.3%), while in the validation set, tenofovir/emtricitabine/DRV (48.2%) and abacavir/lamivudine/DRV (16.5%) made up a majority of ARTs. Genotype frequencies were comparable. Finally, while the validation set only included single concentration data collected at random post-intake times, the learning set also included 12 PK profiles over 6 h (Fig. 1). On average, post-intake times were longer in the validation set.

**Table 1:** Summary characteristics of the learning and validation sets

	Learning set		Validation set		*p*
Patients	127		164		
Samples	405		180		
DRV dosage					< 0.05*
300 mg q12h 600 mg q24h 600 mg q12h 800 mg q24h 900 mg q24h 1200 mg q24h	0 0 10 116 0 1	0% 0% 7.9% 91.3% 0% 0.8%	1 1 48 110 1 3	0.6% 0.6% 29.3% 67.1% 0.6% 1.8%	
Booster					< 0.05*
RTV COB	18 109	14.2% 85.8%	164 0	100% 0%	
Post-intake delay (h)					< 0.05*
Median (range) q24h dosing q24h dosing (sparse only) q12h dosing	6.3 (0.5–29) 6.9 (0.5–29) 12.3 (1–29) 3.8 (2.2–27.5)		14.8 (2–31.5) 16.2 (2.3–31.5) 16.2 (2.3–31.5) 13.7 (2–25.8)		
Age (years)					< 0.05*
Median (IQR)	55 (13)		48 (14)		
Sex					0.62
Male Female	85 42	66.9% 33.1%	105 59	64.0% 36.0%	
Race					0.26
Caucasian African Other	67 55 5	52.8% 43.3% 3.9%	101 60 3	61.6% 36.6% 1.2%	
Concomitant ARVs					< 0.05*
Tenofovir Emtricitabine Lamivudine Abacavir Nevirapine Etravirine Rilpivirine Dolutegravir Raltegravir Maraviroc	35 28 42 8 0 7 7 79 7 21	27.6% 22.0% 33.1% 6.3% 0% 5.5% 5.5% 62.2% 5.5% 16.5%	92 84 46 29 2 18 0 4 43 15	56.1% 51.2% 28.0% 17.7% 1.2% 11.0% 0% 2.4% 26.2% 9.1%	
Drug-drug interactions					0.66
CYP3A inhibitors CYP3A inducers P-glycoprotein inhibitors	3 7 5	2.4% 5.5% 3.9%	8 21 8	6.3% 16.5% 6.3%	
*CYP3A5* g.6986A>G					0.58
*1/*1 *1/*3 *3/*3 Missing	33 33 58 3	26.0% 26.0% 45.7% 2.4%	37 32 77 18	22.6% 19.5% 47.0% 11.0%	
*SLCO3A1* g.91941607G>T					0.73
GG GT TT Missing	105 18 0 4	82.7% 14.2% 0% 3.1%	127 19 0 18	77.4% 11.6% 0% 11.0%	

*ARVs* antiretrovirals

*Statistically significant difference (*p* < 0.05). All covariates given at baseline, except post-intake delay (given for all timepoints)

### External validation

Since AAG was not available in the validation set, this covariate had to be removed from the final model, which led to a 43 points increase in the objective function value (OFV), and this reduced model was validated instead. It was considered preferable to remove AAG entirely because imputation methods could not be easily applied to substitute all the missing values without distorting the AAG distribution that would have been observed, also keeping in mind the possibility of correlation between AAG and other variables. Other than the increase in OFV, this reduced model was very similar to the final model in terms of goodness of fit, with only one individual prediction being noticeably shifted (Online Resource 1 and Online Resource 2).

**Fig. 2:**
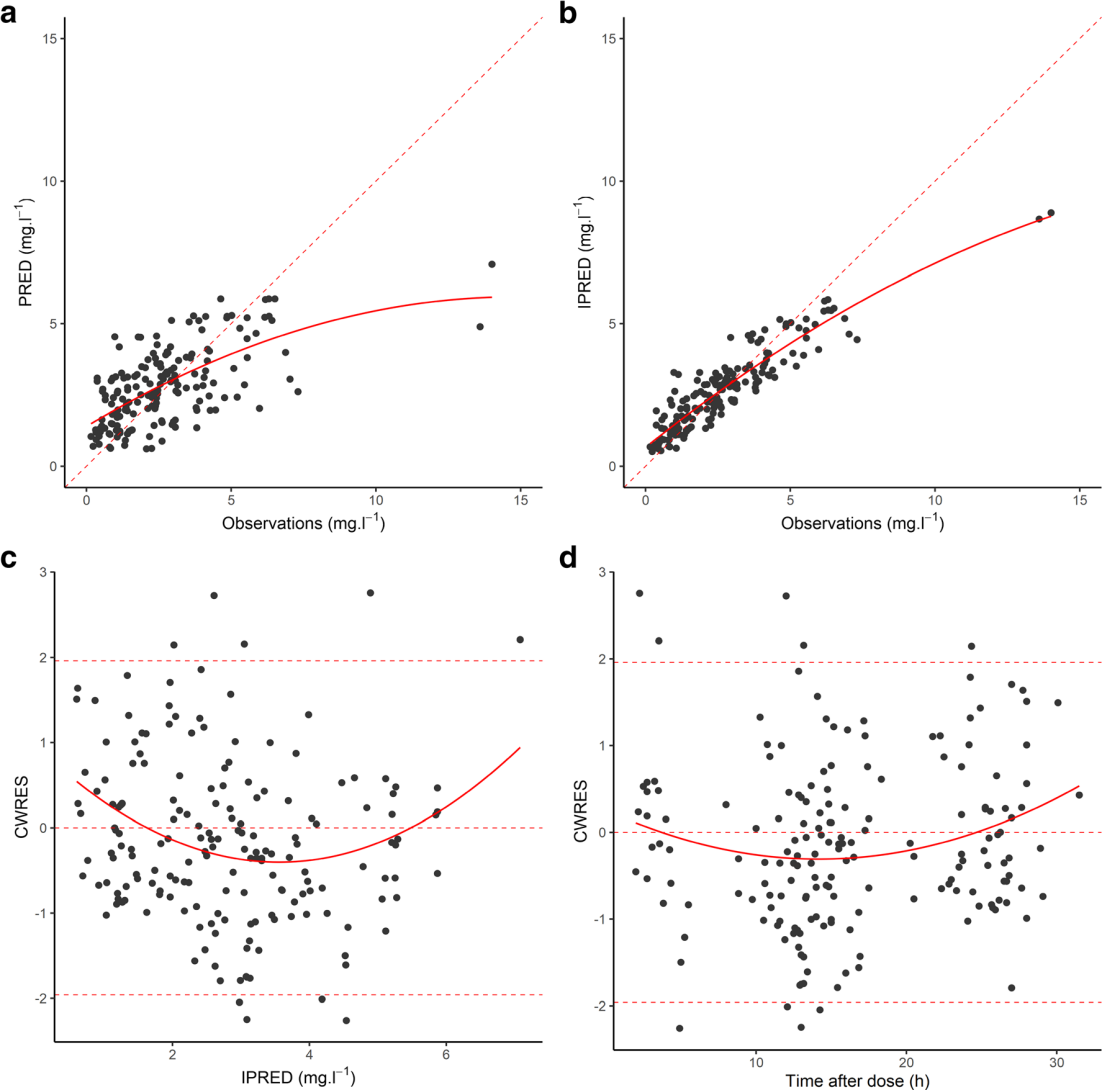
Goodness of fit plots for the validation set. **a** Population predictions (PRED) versus observed concentrations. **b** Individual predictions (IPRED) versus observed concentrations. **c** Conditional weighted residuals (CWRES) versus PRED. **d** CWRES versus time after dose. Dashed line is the line of identity (panels **a** and **b**) or the reference CWRES range assuming a normal distribution (panels **c** and **d**), and continuous line is the LOESS fit line (all panels)

**Fig. 3:**
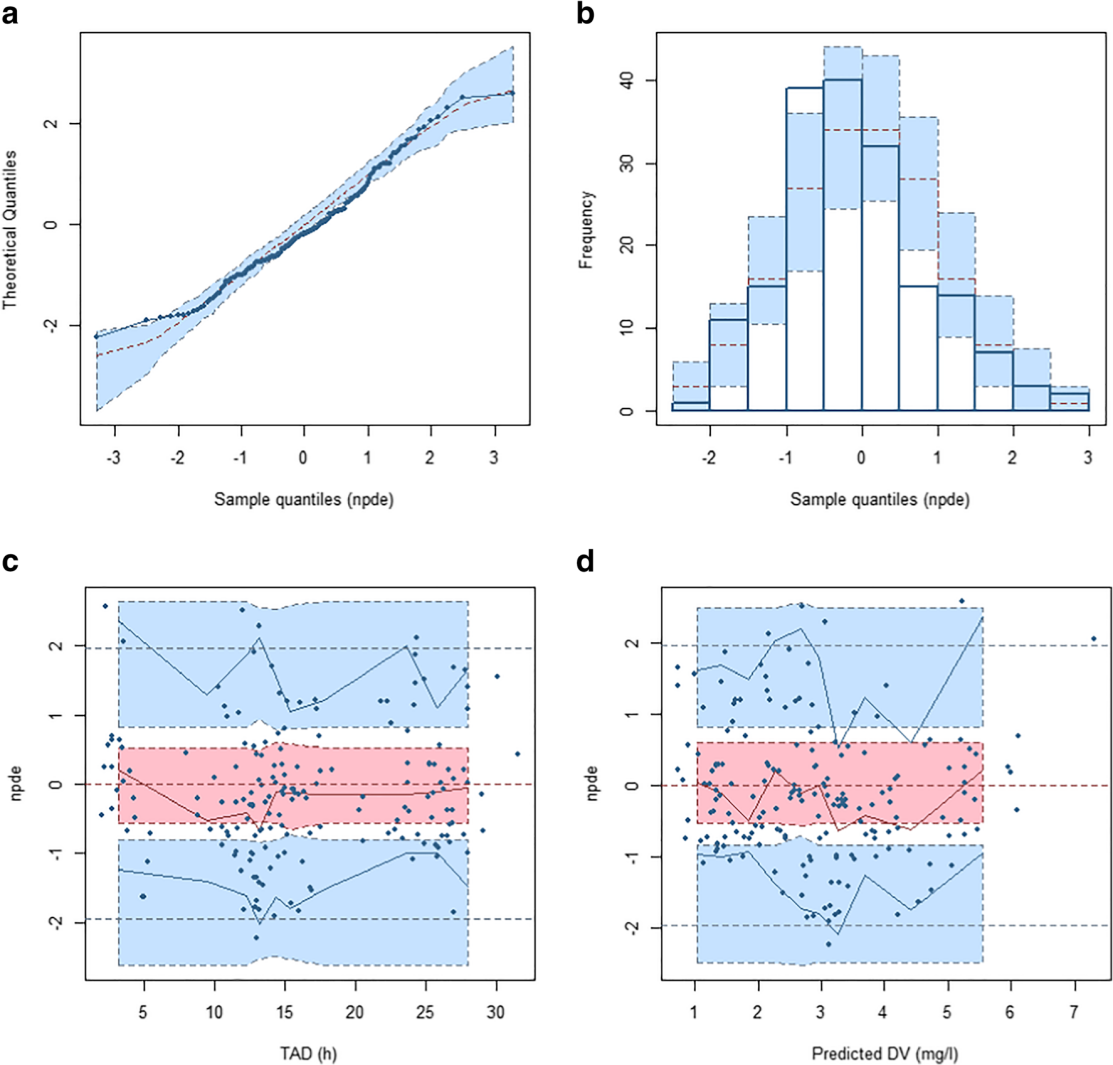
NPDE for the validation set. **a** Q-Q plot. **b** Histogram of NPDE. Theoretical distribution represented as shaded bars. **c** NPDE versus time after dose (TAD). Prediction intervals represented as shaded areas. Observations plotted as circles and observation percentiles as solid lines. **d** NPDE versus PRED

**Fig. 4:**
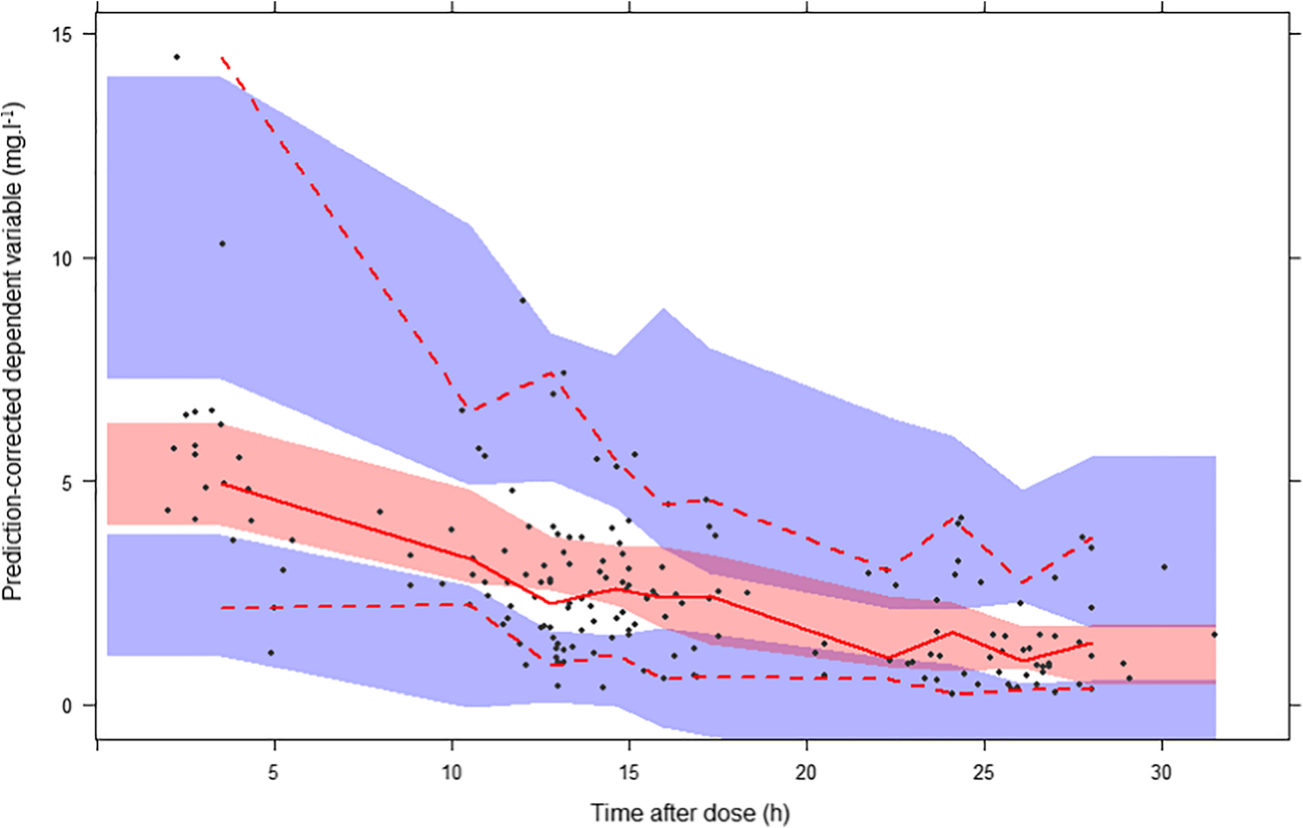
Prediction-corrected visual predictive check for the validation set. Circles represent observed concentrations, solid line is the median of simulations, dashed lines are the 5th and 95th percentiles of simulations, and shaded areas represent CIs around simulation percentiles

Standard goodness of fit plots obtained after fixing all parameters to their final estimates are displayed in Fig. 2. With the exception of two high concentrations (13.6 and 14.01 mg l^−1^) that were severely underpredicted, there was decent agreement between observations and predictions, although trends toward over- and underprediction were noted for the lower and upper ends of the concentration range, respectively. No obvious trend was apparent in the conditional weighted residuals. Meanwhile, NPDE deviated from their expected normal distribution (Fig. 3). The *p* values for the Wilcoxon signed-rank test, Fisher variance test, and the Shapiro-Wilk test were 0.1, 0.39, and 0.01, respectively. Thus, the global adjusted *p* value (accounting for multiple testing) was 0.04. A pcVPC of the final model predictions versus the validation data is presented in Fig. 4: most observations lied within the 95% prediction interval, but due to the low number of data points at higher concentrations, these were characterized by a large uncertainty, especially during the absorption phase.

### Merged data analysis

Model parameters remained similar after re-estimation on the merged learning/validation set (Table 2). Median bootstrapped parameters were comparable, with noticeably large CIs on k_a_ and the effect of *SLCO3A1* genotype on V/F. The difficulty in obtaining accurate estimates of k_a_ can be attributed to the lack of samples in the absorption phase. Goodness of fit was similar to that of the learning set, with a trend toward underprediction of high concentrations and, in the case of NPDE, a departure from normality (Online Resource 3 and Online Resource 4).

**Table 2:** Comparison of model parameters with learning, merged, and bootstrapped data

Parameter	Learning set	Merged set	Bootstrapped data
CL/F (l h^−1^)	12.6	12.4	12.3 [11.1–13.8]
ω_CL_ (sd)	0.238	0.248	0.240 [0.162–0.291]
V/F (l)	137	147	144 [110–170]
ω_V_ (sd)	0.353	0.308	0.292 [0.069–0.427]
k_a_ (h^−1^)	0.545	0.724	0.704 [0.371–1.419]
ω_ka_ (sd)	0.575	0.712	0.826 [0.335–1.762]
θ_sex_ on CL	− 0.198	− 0.151	− 0.153 [− 0.258 to − 0.046]
θ_CYP3A5_ on CL	− 0.192	− 0.126	− 0.131 [− 0.230 to − 0.029]
θ_SLCO3A1_ on V	0.991	0.697	0.756 [0.230–1.848]
σ_exponential_ (sd)	0.306	0.334	0.325 [0.265–0.382]
σ_additive_ (sd)	0.611	0.539	0.548 [0.349–0.715]

Parameters given as fixed or random effect. Bootstrapped parameters given as median [95% CI]. CL/F, apparent clearance; V/F, apparent volume of distribution; k_a_, absorption rate constant; ω, inter-individual variability; σ, residual variability

### Optimal sampling strategy

**Fig. 5:**
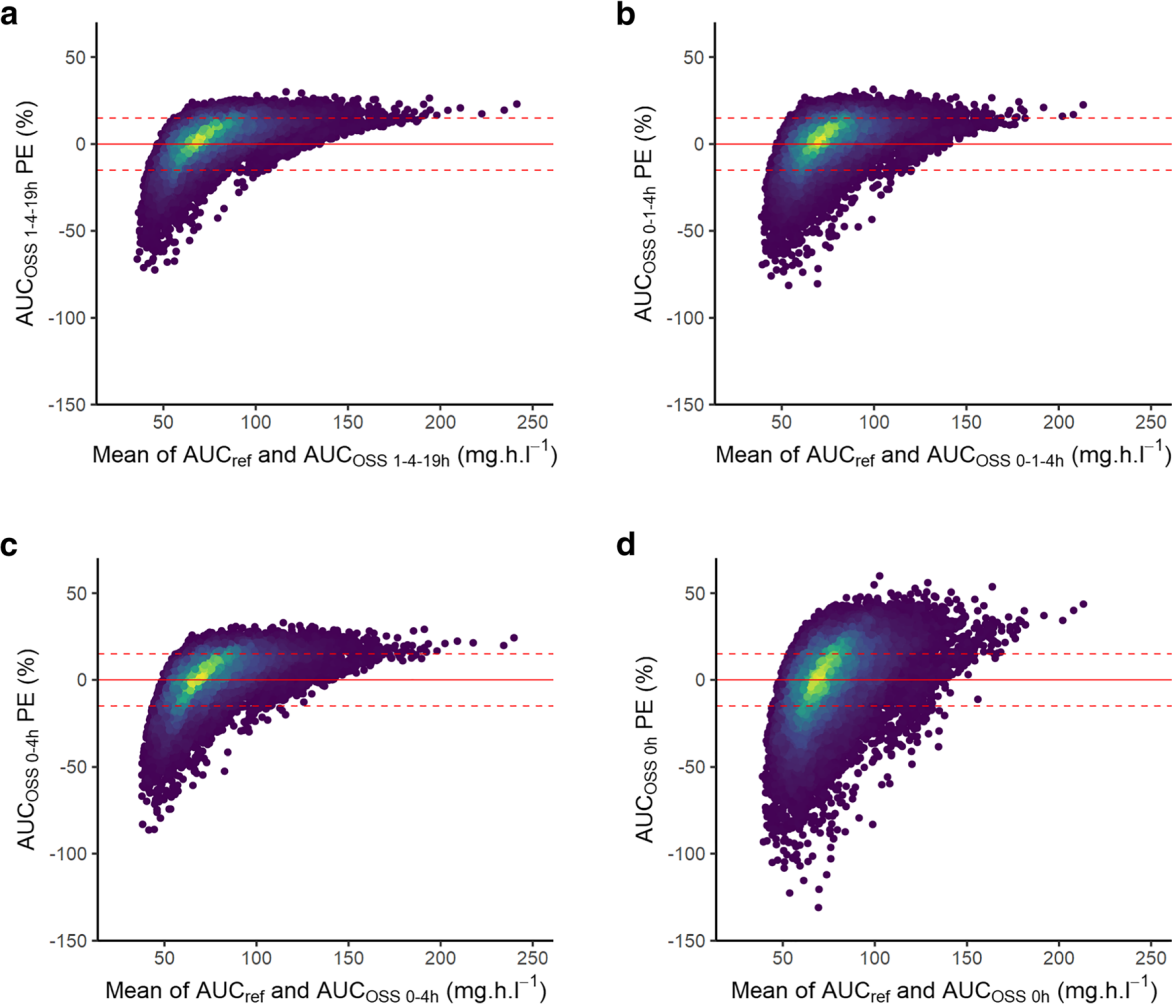
Bland-Altman plots of predicted AUCs for select strategies. Point density indicated by the color scale, from dark (least dense) to light (most dense), with the scale being proper to each panel. **a** C_1_-C_4_-C_19_ (optimal design). **b** C_0_-C_1_-C_4_ (near-optimal design). **c** C_0_-C_4_ (best two-point design). **d** C_0_ (best one-point design)

A design including three points was found to be equivalent to the base design of our clinical study. The D-optimal design included C_1_, C_4_, and C_19_, with a correlation coefficient of 0.93, an MPE of 1.4%, an RMSPE of 12.1%, and 81.2% AUCs predicted with less than 15% prediction error (Table 3). Late post-intake times may not be convenient for sampling, as a patient who usually takes their medication early in the morning would need to either be sampled during the night or change their drug-taking habits; hence, we also assessed the effect of substituting C_19_ by a pre-dose sample (C_0_). The objective functions calculated when evaluating a C_1_-C_4_-C_19_ and C_0_-C_1_-C_4_ design in PopED were comparable (delta = 0.081), and the latter strategy yielded similar results in NONMEM as well (Table 3), showing that the C_19_ may be substituted with a C_0_ if it is more convenient. It was not possible to compute the objective function in PopED for a one- or two-point design, perhaps indicating that these were not sufficiently informative. Regardless, potential one- and two-point OSSs were evaluated in NONMEM by assuming they would include the same times selected for the three-point OSS (i.e., one-point designs were C_0_, C_1_, or C_4_, and two-point designs were C_0_-C_1_, C_0_-C_4_, and C_1_-C_4_). The MPE remained low in all evaluated scenarios, likely due to positive and negative prediction errors canceling each other out (Fig. 5). Meanwhile, the RMSPE was less than 15% for both three-point OSSs, as well as the C_0_-C_1_ and C_0_-C_4_ two-point OSSs. The percentage of AUCs predicted within ± 15% of the reference value increased substantially with the number of samples: from 55 with a C_1_ only to 81.2% with the optimal design (Table 3, Fig. 5). 86.8% of AUC_ref_ within a clinically meaningful range of 50 to 130 mg h l^−1^ (equal to the 95% prediction interval in the learning set) were accurately predicted with the optimal design, while bias grew for AUCs outside of this range for every strategy. Prediction errors did not appear to be correlated with individual covariate values based on visual inspection (data not shown). Lastly, sensitivity analysis showed that estimates of CL, V, and k_a_ in the initial design mostly affected the timing of the last sample, which ranged from 16 to 24 h, depending on the case.

**Table 3:** Comparison of sampling strategies with regard to AUC calculation

Strategy	ρ_AUC_	MPE_AUC_ (%)	RMSPE_AUC_ (%)	AUCs < 15% PE (%)
C_1_-C_4_-C_19_	0.93	1.4	12.0	81.2
C_0_-C_1_-C_4_	0.92	0.7	12.8	79.2
C_0_-C_1_	0.88	− 0.9	14.7	73.2
C_0_-C_4_	0.90	0.4	13.7	75.3
C_1_-C_4_	0.75	0.1	18.8	58.9
C_0_	0.82	− 1.7	17.1	65.0
C_1_	0.69	− 1.5	20.7	55.3
C_4_	0.72	− 0.2	19.7	56.2

## Discussion

Basic goodness of fit metrics and internal validation techniques relying on Monte Carlo simulations are the norm for the validation of population PK models, while external validation is more rarely conducted, despite contributing a useful piece of information [2]. The validation set used for the present work was similar to the learning set in terms of sample size, which allowed us to capture the magnitude of the inter-individual variability that is observed in clinical practice, and thus to assess the robustness of our model over a wide range of concentrations. However, the two datasets differed in several ways. The first major difference was the lack of COB users in the validation set since it predates the introduction of this booster in the therapeutic arsenal. However, the results of the external validation actually show that a model developed mostly on DRV/COB data can reliably be used to predict individual parameters in DRV/RTV users. The second difference was the absence of AAG, which had been used as a covariate in our original model. This meant that external validation had to be performed not on the final model, but on an earlier iteration that did not include AAG. Despite a significant increase in the objective function when this covariate was removed from the model, the goodness of fit remained comparable in terms of visual trends on standard plots, parameter estimates, and precision. PK data from the validation set was also characterized by slightly longer post-intake times, although this was partly due to the presence of twelve 6-h PK profiles in the learning data, which skewed the median. Nevertheless, there were fewer samples available during the early PK phase for that dataset. The last major difference was in the ARV backbone and concomitant medications. Since most coadministered ARVs are not known to cause major PK interactions with DRV, disparities in ART should not be an issue as far as model predictions are concerned [18]. The only exception is etravirine, which was the perpetrator drug in all cases of CYP induction in the learning set and in 86% of cases in the validation set (followed by nevirapine, 9.5%, and amiodarone, 4.8%), and which was used twice as often in the validation set compared to the learning set. Although the model did not incorporate a drug interaction component, the number of subjects with potentially induced CYP metabolism was low, and their individual predictions did not appear biased.

After fixing model parameters, the goodness of fit was re-assessed with the validation data. General trends were captured well, but concentrations at the upper end of the observed range (≥ 10 mg l^−1^) were poorly predicted. This model misspecification may have been due to the lack of data at high concentrations or the lack of a key covariate that could explain these outliers. Simulation-based diagnostics such as the NPDE and VPC yielded more nuanced results, showing moderate deviation from the reference normal distribution in the case of NPDE, and large CIs in the case of the VPC. The large CIs could be due to the heterogeneous sampling design, as few samples were collected in certain time ranges, which also made it difficult to find an appropriate binning strategy. Meanwhile, parameter estimates remained almost unchanged by the addition of the validation set to the learning set, confirming model robustness. In any event, this analysis highlights the usefulness of simulation-based diagnostics in assessing model performance.

Using this model, we then evaluated limited sampling strategies for DRV, which could be used to enhance TDM or to design further PK studies. To our knowledge, no such strategies have been proposed in the literature. Should the clinician only need a rough idea of patient adherence, a single C_0_ can readily be used and interpreted according to recommended levels for PI-naïve or PI-pretreated patients. However, due to the important inter-individual variability that exists for DRV, this C_0_ may be difficult to interpret, and low concentrations would not necessarily be indicative of low adherence. Furthermore, if a C_0_ cannot be obtained at the time of TDM, a population model may be used to extrapolate it from available information or to derive a more complete measurement of drug exposure, which could for instance be used to assess whether the patient is eligible for alternative dosage regimens or to investigate possible causes of under- or over-exposure. The model we previously proposed is applicable to any HIV patient under DRV-based therapy, regardless of concomitant antiretrovirals, but requires AAG measurement and genotyping for two single nucleotide polymorphisms. In case additional laboratory costs and analysis time are an issue, a model without these covariates may also be used without a significant loss of predictive power, as we have shown in the present paper by validating a model which did not feature AAG. In any event, to obtain a precise estimate of individual PK parameters, samples should be as informative as possible—this is where an OSS can come in useful. Different strategies were evaluated using D-optimal design and keeping practical limitations in mind. While it is possible to first derive an OSS and to then evaluate it on a new validation set by comparing the parameters one obtains using the entire validation set with those obtained using only the previously determined optimal times, this requires the validation data to include all points selected in the OSS. This was unfortunately not the case here, as only sparse concentrations were collected in our patients, whereas the OSS included multiple points per individual. Instead, a simulation approach was used: AUCs were simulated based on the learning set (once again, the validation set could not be used due to the lack of AAG), and we assessed how accurately this parameter could be predicted using each strategy of interest. The AUC was chosen as the parameter to be estimated because it reflects total exposure and it (or CL) is the parameter one would mostly be interested in predicting, although, from a clinical point of view, it has yet to be convincingly demonstrated that AUC is a better marker of exposure than the C_0_ or, for that matter, that there is a correlation between DRV exposure and efficacy [1, 19, 20]. If only a single sample can be obtained, C_0_ was found to be the best choice. The two-point OSS was C_0_ and C_1_, while the three-point OSS was C_0_, C_1_, and C_4_. Although C_1_-C_4_-C_19_ was initially found to be the optimal strategy, sensitivity analysis showed some variation in the timing of the last sample depending on input parameters, but using a C_0_ instead did not appear to have much influence, and a C_0_ is likely the most convenient option for clinicians. All three OSSs were found to be adequate in terms of bias and precision, but extreme values of AUC were more poorly predicted with sparser designs (although AUCs within a clinically relevant range were more often predicted accurately). The three-point design, while imperfect, provided the most information over a larger range of AUCs.

## Conclusion

A previously published population PK model for DRV was validated using an external dataset. Results confirm its robustness and lend more credibility to model-derived predictions. Several sampling strategies were evaluated, among which a three-point strategy (either C_0_, C_1_, and C_4_ or C_1_, C_4_, and C_19_) was the most adequate. These strategies could be used as a starting point for the design of further PK studies in order to maximize the amount of information that can be gathered, especially in resource constrained settings.

## Supplementary information

ESM 1 (DOC 1092 kb)

## Data Availability

The datasets generated during and/or analyzed during the current study are available from the corresponding author on reasonable request.

## Abbreviations

AAG: alpha-1-acid glycoprotein
ART: antiretroviral therapy
ARV: antiretroviral
AUC: area under the curve
BFGS: Broyden-Fletcher-Goldfarb-Shanno
C_0_: trough concentration
COB: cobicistat
CWRES: conditional weighted residuals
DRV: darunavir
IPRED: individual prediction
MPE: mean percentage error
NPDE: normalized prediction distribution error
OFV: objective function value
OSS: optimal sampling strategy
PE: prediction error
PI: protease inhibitor
PK: pharmacokinetic
PRED: population prediction
RMSPE: root mean square percentage error
TDM: therapeutic drug monitoring
(pc)VPC: (prediction-corrected) visual predictive check
