# Supplementary material for: Optimal sampling strategies for darunavir and external validation of the underlying population pharmacokinetic model
Source: Eur J Clin Pharmacol. 2020 Nov 11;77(4):607–16. doi: 10.1007/s00228-020-03036-2 (PMC7935830; doi:10.1007/s00228-020-03036-2)
Supplement: Supplementary file 1 — (DOC 1092 kb) [file 228_2020_3036_MOESM1_ESM.doc]

**Optimal sampling strategies for darunavir and external validation of the underlying population pharmacokinetic model**

Gabriel Stillemans^1,2*^, Leila Belkhir^2,3^, Bernard Vandercam^3^, Anne Vincent^3^, Vincent Haufroid^2,4^, Laure Elens^1,2^

^1^ Integrated PharmacoMetrics, PharmacoGenomics and PharmacoKinetics, Louvain Drug Research Institute, Université catholique de Louvain, Brussels, Belgium

^2^ Louvain centre for Toxicology and Applied Pharmacology, Institut de recherche expérimentale et clinique, Université catholique de Louvain, Brussels, Belgium

^3^ AIDS Reference Center, Department of Internal Medicine, Cliniques universitaires Saint-Luc, Université catholique de Louvain, Brussels, Belgium

^4^ Department of Clinical Chemistry, Cliniques universitaires Saint-Luc, Brussels, Belgium

*Corresponding author

Postal address: Avenue E. Mounier 72, B01.72.02, Brussels, Belgium

Phone number: +32 2 764 72 97

e-mail: [gabriel.stillemans@uclouvain.be](mailto:gabriel.stillemans@uclouvain.be)

ORCID

GS: 0000-0002-2356-4891

LB: 0000-0002-1701-7584

VH: 0000-0001-5040-9806

LE: 0000-0002-0039-3583

# DECLARATIONS

**Funding**

**This work was supported by the Fonds pour la Formation à la Recherche dans l’Industrie et dans l’Agriculture (FRIA) (grant number FC16749 to Gabriel Stillemans).**

**Conflicts of interest/Competing interests**

**All authors declare that they have no conflict of interest.**

**Ethics approval**

Not applicable.

**Consent to participate**

**Not applicable.**

**Consent for publication**

Not applicable.

**Availability of data and material**

**The datasets generated during and/or analyzed during the current study are available from the corresponding author on reasonable request.**

**Code availability**

**Not applicable.**

### Supplementary Material 1. Effect of AAG removal on model fit

|  | **Full model** | | | **Reduced model** | | |
| --- | --- | --- | --- | --- | --- | --- |
| **OFV** | 617.307 | | | 660.683 | | |
| **Structural model** | **Estimate** | **RSE (%)** | **Shrinkage (%)** | **Estimate** | **RSE (%)** | **Shrinkage (%)** |
| CL/F (l.h^-1^)  ω_CL_ (SD) | 12.9  0.22 | 5.3  14.0 | 33.3 | 12.6  0.24 | 5.5  14.1 | 31.7 |
| V/F (l)  ω_V_ (SD) | 152  0.33 | 21.1  16.4 | 59.0 | 137  0.35 | 16.3  17.1 | 56.6 |
| k_a_ (h^-1^)  ω_ka_ (SD) | 0.68  0.60 | 73.5  22.3 | 63.1 | 0.55  0.58 | 43.5  22.3 | 62.7 |
| **Covariate model** |  | | |  | | |
| AAG on CL  AAG on V  Female sex on CL  *SLCO3A1 G>T* on V  *CYP3A5*3* on CL | -0.61  -0.68  -0.21  0.81  -0.16 | 37.9  26.3  27.3  59.5  35.5 |  | /  /  -0.20  0.99  -0.19 | /  /  30.0  46.1  28.3 |  |
| **Residual variability** |  | | |  | | |
| σ_exponential_ (SD)  σ_additive_ (SD) | 0.281  0.641 | 13.9  19.7 | 11.5  11.5 | 0.306  0.611 | 13.4  22.9 | 11.7  11.7 |

CL/F: Apparent clearance, k_a_: Absorption rate constant, OFV: Objective function value, RSE: Relative standard error (= standard error/parameter absolute value), V/F: Apparent volume of distribution, ω: Random effect (inter-individual variability), σ: Random effect (residual variability).

###
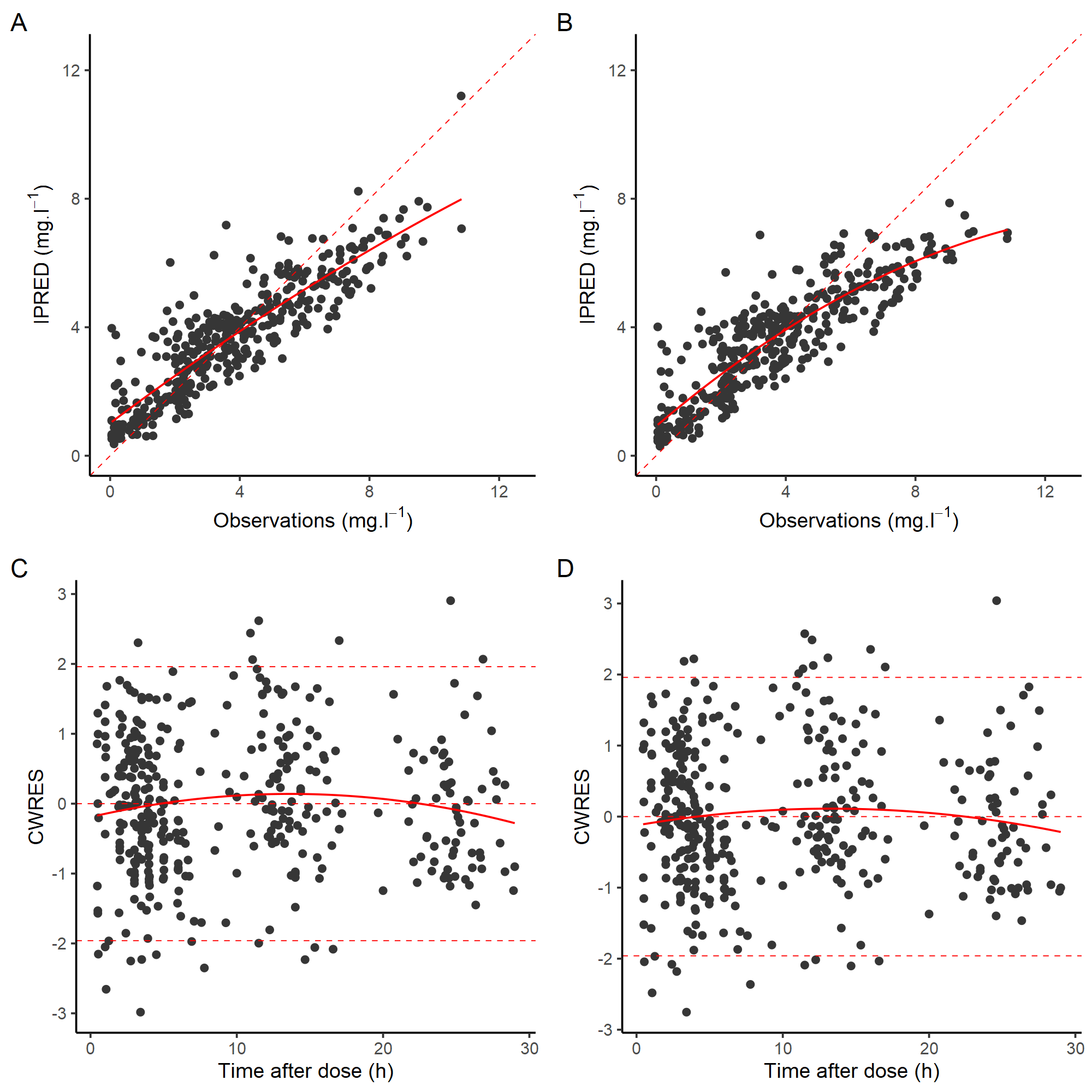


**Figure S1.** Goodness of fit after removal of AAG. (**A**) Individual predictions (IPRED) versus observed concentrations, full model. (**B**) IPRED versus observed concentrations, reduced model. (**C**) Conditional weighted residuals (CWRES) versus time after dose, full model. (**D**) CWRES versus time after dose, reduced model. Dashed red line is the line of identity (panels **A** and **B**) or the reference CWRES range assuming a normal distribution (panels **C** and **D**), continuous red line is the LOESS fit line (all panels).

### **Supplementary Material 2.** Goodness of fit for merged dataset


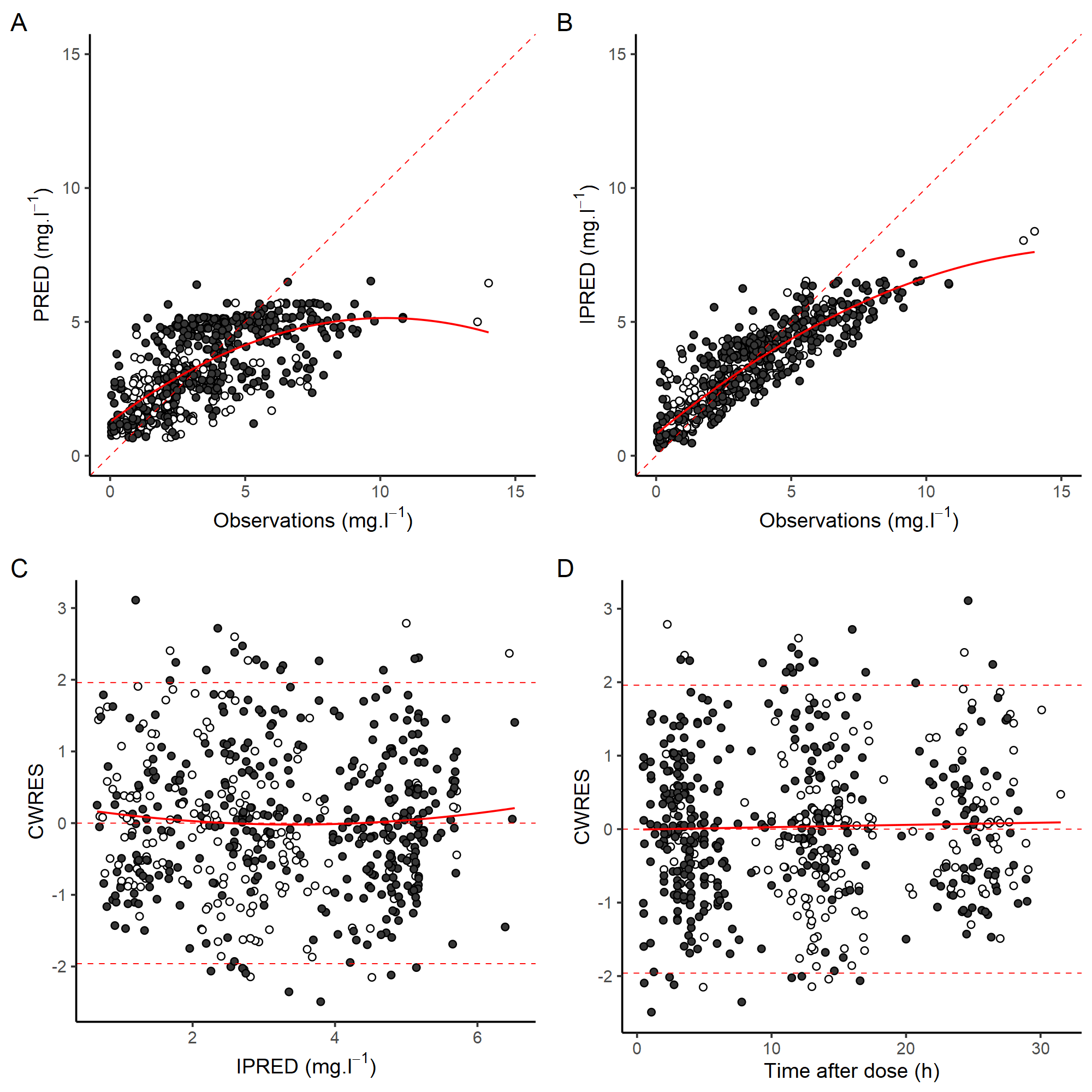


### Figure S2. Goodness of fit plots for merged dataset. (**A**) Population predictions (PRED) versus observed concentrations. (**B**) Individual predictions (IPRED) versus observed concentrations. (**C**) Conditional weighted residuals (CWRES) versus PRED. (**D**) CWRES versus time after dose. Learning set represented with black circles, validation set with white circles. Dashed red line is the line of identity (panels **A** and **B**) or the reference CWRES range assuming a normal distribution (panels **C** and **D**), continuous red line is the LOESS fit line (all panels).


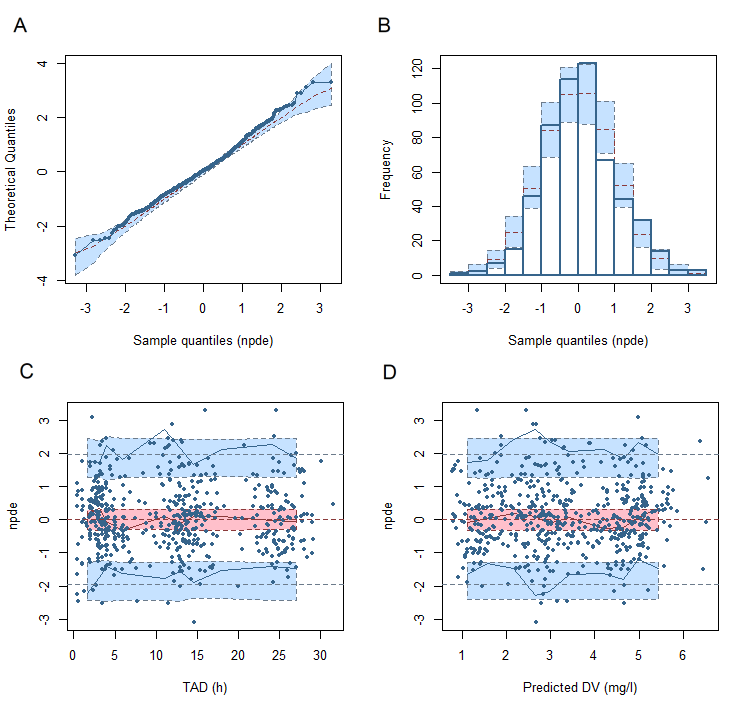


**Figure S3.** NPDE for merged dataset. (**A**) Q-Q plot. (**B**) Histogram of NPDE. **Shaded area represents theoretical distribution.**  (**C**) NPDE versus time after dose (TAD). **Shaded areas represent the prediction intervals associated with the 5^th^, 50^th^ and 95^th^ percentiles.** (**D**) NPDE versus PRED.

| **Test** | **p-value** |
| --- | --- |
| Wilcoxon signed-rank test | 0.0853 |
| Fisher variance test | 0.674 |
| Shapiro-Wilk test | 0.0106* |
| Global adjusted | 0.0317* |

* p-value < 0.05
